# A highly charged region in the middle domain of plant endoplasmic reticulum (ER)-localized heat-shock protein 90 is required for resistance to tunicamycin or high calcium-induced ER stresses

**DOI:** 10.1093/jxb/eru403

**Published:** 2014-10-08

**Authors:** Lisa P. Chong, Yao Wang, Nanette Gad, Nathaniel Anderson, Bhavank Shah, Rongmin Zhao

**Affiliations:** Department of Biological Sciences, University of Toronto, Toronto, Ontario, CanadaM1C 1A4

**Keywords:** Enzyme kinetics, ER stress responses, HSP90 client proteins, molecular chaperone, protein–protein interaction, transgenic plant.

## Abstract

Heat-shock protein 90 (HSP90) is a highly conserved molecular chaperone that is involved in modulating a multitude of cellular processes under both physiological and stress conditions. In *Arabidopsis*, there are seven HSP90 isoforms (HSP90.1–HSP90.7) that are localized in the cytoplasm/nucleus, mitochondrion, chloroplast, and endoplasmic reticulum (ER) where protein folding actively takes place. In this study, we analysed the sequence of ER-localized *Arabidopsis* HSP90.7 and the other ER GRP94 proteins from plants and animals, and identified a short, charged region that is specifically present in the middle domain of plant-derived GRP94 proteins. To understand the role of this charged region, we analysed transgenic plants that expressed a mutant protein, HSP90.7^Δ22^, which had this charged region deleted. We showed that seedlings expressing HSP90.7^Δ22^ had significantly enhanced sensitivity to ER stress induced by tunicamycin or a high concentration of calcium, although its general chaperone activity in preventing the model protein from heat-induced aggregation was not significantly affected. We also analysed the ATP-binding and hydrolysis activity of both wild-type and mutant HSP90.7 proteins, and found that they had slightly different ATP-binding affinities. Finally, using a yeast two-hybrid screen, we identified a small set of HSP90.7 interactors and showed that the charged region is not required for the candidate client interaction, although it may affect their binding affinity, thus providing potential targets for further investigation of HSP90.7 functions.

## Summary

Plant ER-localized HSP90 contains a conserved and charged fragment, which is required for calcium- or tunicamycin-induced ER stress resistance, and deletion of the fragment affects the protein ATPase activity.

## Introduction

The heat-shock protein 90 (HSP90) family are well-conserved molecular chaperones in both prokaryotes and eukaryotes; they have a global role in maintaining cellular protein homeostasis and participate in a wide range of cellular processes. HSP90, in particular, is well documented in its essential role in folding a variety of protein kinases and steroid hormone receptors (Richter and Buchner, 2001; Young *et al.*, 2001). HSP90 is also required for the assembly of multiple macromolecular structures (Makhnevych and Houry, 2012), for buffering genetic variations in *Arabidopsis* (Sangster *et al.*, 2008), *Drosophila* (Rutherford and Lindquist, 1998), and fungi (Cowen and Lindquist, 2005), and for plant pathogen-related disease resistance (Lu *et al.*, 2003; Takahashi *et al.*, 2003; Boter *et al.*, 2007).

HSP90 is structurally divided into three domains, an ATP-binding N-terminal domain (Stebbins *et al.*, 1997), a substrate-binding middle domain (Sato *et al.*, 2000), and a C-terminal dimerization domain, which allows two HSP90 monomers to associate and form an active HSP90 protein (Minami *et al.*, 1994). In addition, a highly charged glutamic acid-rich linker region is found between the N-terminal and the middle domains, and has been shown to facilitate client protein activation (Hainzl *et al.*, 2009). The HSP90 family can be further divided into five subfamilies based on their subcellular localization, the cytosolic HSP90A, endoplasmic reticulum (ER)-localized HSP90B/GRP94, chloroplast-localized HSP90C, mitochondria-localized TRAP (tumor necrosis factor receptor-associated protein), and bacterial HSP90, which is also known as HtpG (high-temperature protein G) (Taipale *et al.*, 2010). Extensive studies have revealed the evolution, structure, mechanism of action, and complex functional regulation of this protein family (Cowen and Lindquist, 2005; Pearl and Prodromou, 2006; Sangster *et al.*, 2008; Taipale *et al.*, 2010; Johnson, 2012; Kadota and Shirasu, 2012). However, most studies focus on cytosolic HSP90 isoforms, and the structure and regulation of the organelle-localized HSP90s in eukaryotic cells are less well-understood.

In *Arabidopsis thaliana*, seven HSP90 members have been identified and named as HSP90.1–HSP90.7 (Krishna and Gloor, 2001). HSP90.7 is an ER-localized HSP90 paralogue and belongs to the HSP90B subfamily (Chen *et al.*, 2006). ER-localized HSP90 is also known as glucose-regulated protein 94 (GRP94), or gp96 in mammalian cells, and facilitates the folding of many proteins such as IgG, Toll-like receptors, and subclasses of integrins (Marzec *et al.*, 2012). ER-localized HSP90 members are also known as endoplasmin (Csermely *et al.*, 1995). *Arabidopsis* HSP90.7 (we refer to it as HSP90.7 rather than HSP90B or AtGRP94 in this study), has also been called SHEPHERD (SHD) in a previous study for its role in helping CLAVATA (CLV) proteins form a regulatory complex (Ishiguro *et al.*, 2002). A *shd* mutant, which contains a T-DNA insertion in the promoter region of *HSP90.7*, has an enlarged dome-shaped shoot apical meristem, as well as expanded floral meristems (Ishiguro *et al.*, 2002). The *shd* mutant is phenotypically indistinguishable from *clv* mutants, and the *wuschel* (*wus*) mutation was found to be completely epistatic to the *shd* mutation. HSP90.7 is therefore implicated in the correct folding of CLAVATA proteins (comprising CLV1, CLV2, and CLV3), which participate in shoot apical meristem maintenance (Miwa *et al.*, 2009; Aichinger *et al.*, 2012; Somorjai *et al.*, 2012). Additionally, in a study using tunicamycin to induce ER-specific stress on tobacco protoplasts, HSP90.7 was shown to have a role in supporting α-amylase secretion (Klein *et al.*, 2006). However, whether or not HSP90.7 binds directly to α-amylase and the other candidate client proteins that require HSP90.7 function are still unknown. Moreover, analysis of the *shd* mutant also showed that HSP90.7 may not act as a general chaperone to bind as many newly synthesized polypeptides as the HSP70 family chaperone binding immunoglobulin protein (BiP) in the ER, and that HSP90.7 functions specifically in proliferating tissues (Klein *et al.*, 2006).

In this study, we analysed the sequence and function of *Arabidopsis* HSP90.7 and showed that HSP90.7 contains a plant-specific, highly charged 22 aa fragment in the middle domain. By analysing transgenic seedlings that expressed an HSP90.7 mutant that had the charged region deleted, we showed that this region in the middle domain is essential for seedlings to resist ER stress induced by tunicamycin or a high concentration of Ca^2+^. However, the general chaperone activity in preventing model proteins from heat-induced aggregation was not affected by deletion of this charged region. Further biochemical and proteomics analyses of the mutant protein indicated that the charged region might be involved in regulating HSP90.7 ATP-hydrolysis efficiency, and not in directly binding substrate proteins.

## Materials and methods

### Plant materials and growth conditions

The *A. thaliana* ecotype Columbia (Col-0) was used as the wild-type plant. To select for transgenic plants or test plant resistance to abiotic stresses, seeds were surface sterilized and sown on ½-strength Murashige and Skoog (MS; Murashige & Skoog, 1962) medium containing 1% sucrose and 0.7% agar with or without supplementation by abiotic stress-inducing reagents. After stratification in the dark at 4 °C for 3–4 d, the seeds were cultured within a plant growth incubator set at 120 µmol m^–2^ s^–1^ with a 16/8h light/dark cycle at 22 °C. Alternatively, sterilized seeds were stratified in microcentrifuge tubes and then placed over freshly prepared Pro-mix PGX® soil for growth within a plant growth chamber set at 110 µmol m^–2^ s^–1^ with a 16/8h light/dark cycle at 22 °C.

### Construction of the HSP90.7^Δ22^ deletion mutant

Two *Spe*I restriction sites (5ʹ-ACTAGT-3ʹ) were inserted into the Arabidopsis *HSP90.7* coding sequence at 1462 and 1528bp by site-directed mutagenesis using primer 5ʹ-CTTGCTGAAGAAGAT CCTACTAGTG ATGAAATC CATGATGAT-3ʹ with its reverse complement, and primer 5ʹ-AACGAT GAGAAG AAGGGTT AAACTA GTCAATA CACAAA ATTCTGG-3ʹ with its reverse complement, respectively, in the *Escherichia coli* expression vector p11 (Savchenko *et al.*, 2003). The HSP90.7 D488–G509 coding sequence was deleted by cleavage with *Spe*I and religation, and the resulting HSP90.7 mutant was designated HSP90.7^Δ22^. The full-length HSP90.7 and HSP90.7^Δ22^ coding sequences were then cloned into binary vectors pGWB402Ω and pGWB502Ω (Nakagawa *et al.*, 2007), respectively, using Gateway cloning, generating pGWB402Ω-AtHSP90.7 and pGWB502Ω-AtHSP90.7^Δ22^, in which a FLAG tag coding sequence was also inserted at the C terminus before the terminal ER-retention sequence KDEL.

### *Arabidopsis* transformation and screening of transgenic plants

*A. tumefaciens* GV3101 carrying pGWB402Ω-AtHSP90.7 or pGWB502Ω-AtHSP90.7^Δ22^ plasmid was used to transform *Arabidopsis* Col-0 by the floral dip method (Clough and Bent, 1998). Selection of transgenic plants was performed on ½ strength MS medium with 1% sucrose supplemented with 25 µg ml^–1^ of kanamycin for HSP90.7 transgenic plants, or 20 µg ml^–1^ of hygromycin for HSP90.7^Δ22^ transgenic plants. PCR amplification and immunoblotting with anti-FLAG antibody (Sigma) were used to confirm the presence of the transgenes and expression of the FLAG-tagged proteins, respectively.

### HSP90 protein expression and purification

The construct for His_6_-tagged canine GRP94 (73–754Δ41) in pET15b (Dollins *et al.*, 2007) was a kind gift from Daniel Gewirth (Hauptman-Woodward Medical Research Institute, Buffalo, USA). *Saccharomyces cerevisiae* cytosolic HSP82, *Arabidopsis* cytosolic HSP90.2, and the predicted mature forms of ER-localized HSP90.7 (R73–L823) and HSP90.7^Δ22^ were cloned into p11. Constructs were introduced into *E. coli* BL21(DE3)-pRIL (Stratagene), and protein expression was induced by 1mM isopropyl β-d-1-thiogalactopyranoside. His_6_-tagged proteins were purified using Ni-NTA resin (Qiagen), and dialysed overnight. His_6_-tag was cleaved with tobacco etch virus protease and removed by Ni-NTA resin. Size-exclusion chromatography with a Superdex 200 column (GE Healthcare) was used to further purify the proteins. Fractions containing the native purified protein were stored at –80 °C in buffer [25mM Tris/HCl (pH 7.5), 150mM KCl, 10% glycerol, 0.5mM dithiothreitol (DTT)] until further use.

### Abiotic stress-resistance tests

Homozygote line seeds screened from lines that contained a single transgene locus based on antibiotic selection were germinated on ½ strength MS medium for 6 d before being subjected to heat-shock treatment at 45 °C for 5, 10, or 30min. The seedlings were then allowed to recover at 22 °C with a 16/8h light/dark photoperiod. Survival rate was measured by the percentage of seedlings that had at least one green leaf 6 d after recovering from the heat shock. To test ER-specific stress resistance, seeds were germinated on ½ strength MS medium containing tunicamycin (0.3 or 0.4 μg ml^–1^) for 2 or 3 d and then transferred back on to normal ½ strength MS medium for recovery.

### *In vitro* chaperone activity assay

Citrate synthase (CS; Sigma) was dialysed in 20mM HEPES/KOH (pH 7.5), 150mM KCl, and 10mM MgCl_2_ before being used for the heat-induced aggregation assay. CS (500nM) was prepared in a final volume of 150 μl of 20mM HEPES/KOH (pH 7.5) and 2.8mM β-mercaptoethanol with different amounts of HSP90 proteins. The mixtures were added to a 96-well microplate and heated at 45 °C. Light scattering at 340nm was monitored at 45 °C in a Synergy 4 spectrophotometer (BioTek). Control measurements were performed with purified HSP90 protein alone in the absence of CS.

### ATP–Sepharose binding assay

An ATP–Sepharose binding assay was performed as described previously (Hernandez *et al.*, 2002) with some modifications. Purified HSP90 protein (10 μg) was incubated with 15 μl of γ-linked ATP–Sepharose beads (Innova Biosciences) in 225 μl of binding buffer [10mM Tris/HCl (pH 7.5), 50mM KCl, 20mM MgCl_2_, 2mM DTT, 20mM Na_2_MoO_4_, 0.01% Nonidet P-40]. The mixture was incubated for 30min at 30 °C with gentle shaking. The unbound fraction was collected after centrifugation, and the beads were washed four times with binding buffer. The bound protein was eluted from the beads by heating at 100 °C in SDS Laemmli buffer. Equivalent amounts of sample were separated by SDS-PAGE and visualized by silver staining.

### ATP-hydrolysis assay

ATP-hydrolysis activity was measured using a coupled NADH method as described previously (Norby, 1988). The decrease in NADH absorbance at 340nm was measured using a Synergy 4 microplate reader (BioTek). Briefly, for each assay, about 50–100 μg of purified HSP90 protein was used in the following reaction mixture: 25mM HEPES (pH 7.5), 5mM MgCl_2_, 400mM KCl, 0.03% Tween 20, 10% glycerol, 200 μM NADH, 3mM phosphoenol pyruvate, 15.7U of pyruvate kinase (Sigma), and 24.5U of lactate dehydrogenase (Sigma). ATP was added in varying concentrations to determine the *K*_m_ values. For all assays, geldanamycin (National Institutes of Health) was added to a final concentration of 10 μM to measure geldanamycin-sensitive HSP90 activities. The turnover rate (*k*_cat_), binding affinity (*K*_m_), and catalytic efficiencies were analysed using the Michaelis–Menten function in the statistical program GraphPad Prism.

### Yeast two-hybrid screening

The HSP90.7 middle and C-terminal domains (HSP90.7^332–823^), designated HSP90.7MC, were cloned into the pEG202 vector for yeast two-hybrid screening. An *Arabidopsis* cDNA library constructed into prey vector pJG4-5 was a kind gift from Dr Gazzarrini (University of Toronto, Canada), and the screening of positive interaction using *LEU2* and *lacZ* marker genes was performed in *S. cerevisiae* EGY48 cells as described previously (Tsai and Gazzarrini, 2012). Positive hits were sequenced and retransformed back to yeast cells to test interactions with wild-type HSP90.7MC, the deletion mutant HSP90.7^Δ22^MC, and the bicoid-bait negative-control RFMH1 (Manseau *et al.*, 1996).

### Antibodies

Polyclonal rabbit anti-HSP90.7 antibody was generated by Signalway AntiBody (College Park, USA) with purified HSP90.7 protein. The other primary antibodies used in this study were anti-FLAG monoclonal antibody (F3165; Sigma) and polyclonal anti-HA antibody (Cedarlane), anti-BiP antibody (Santa Cruz), anti-calnexin antibody (a kind gift from Dr David Williams, University of Toronto, Canada), and anti-HSP90.2 antibody, which was originally raised using purified *S. cerevisiae* HSP82 (Zhao *et al.*, 2005) but also specifically recognizes *Arabidopsis* cytosolic HSP90.1 and HSP90.2 but not the other organelle forms of HSP90 (Song *et al.*, 2009*b*).

## Results

### Plant ER-localized molecular chaperone HSP90 contains a highly charged region in the middle domain

**Fig. 1.**
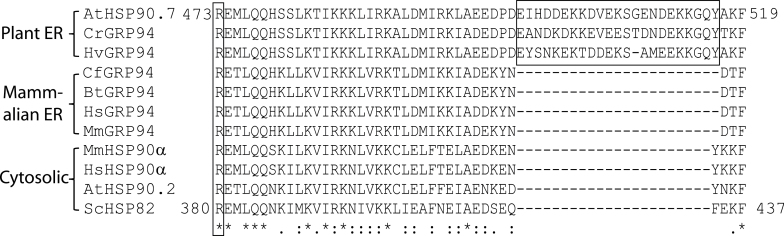
The middle domains of plant ER-localized HSP90 homologues contain a highly charged fragment. Alignment of amino acid sequences surrounding the *Arabidopsis* HSP90.7 middle domain with those from other HSP90 homologues. The highly charged regions specific to plant ER-localized HSP90 are highlighted in a rectangular box. Identical amino acids are indicated by ‘*’, and similar or strongly similar amino acids are indicated by ‘.’ or ‘:’, respectively, under the sequences. AtHSP90.7: *Arabidopsis thaliana* (UniProtKB no. Q9STX5); CrGRP94: *Catharanthus roseus* (P35016); HvGRP94: *Hordeum vulgare* (P36183); CfGRP94: *Canis familiaris* (P41148); BtGRP94: *Bos taurus* (Q95M18); HsGRP94: *Homo sapiens* (P14625); MmGRP94: *Mus musculus* (P08113); MmHSP90α: *Mus musculus* (P07901); HsHSP90α: *Homo sapiens* (P07900); AtHSP90.2: *Arabidopsis thaliana* (P55737); ScHSP82: *Saccharomyces cerevisiae* (P02829).

To understand whether the ER-localized *Arabidopsis* HSP90.7 had any special structural features, we aligned it with other ER-localized HSP90 isoforms from plant and mammalian species, as well as with representative cytosolic HSP90 members. HSP90.7 contained all conserved amino acids for ATP binding (Supplementary Fig. S1 at *JXB* online) in the N terminus and the γ-phosphate-binding amino acid Arg (Pearl and Prodromou, 2006) in the middle domain (Fig. 1). The mammalian GRP94 contains a ‘DGQST’ motif that is close to the ATP-binding site and forms an *N*-ethylcarboxamidoadenosine-binding pocket in the N terminus (Soldano *et al.*, 2003). However, HSP90.7 and the other plant ER-localized GRP94 homologues did not contain such a motif and resembled the cytosolic HSP90 homologues (Supplementary Fig. S1). Surprisingly, HSP90.7 contained a highly charged, 22 aa sequence (D488–G509) in the middle domain, and similar charged fragments were also present in ER-localized HSP90 isoforms from barley (*Hordeum vulgare*) and periwinkle (*Catharanthus roseus*) (Fig. 1). Further analysis indicated that all 11 available ER-localized HSP90 sequences identified from the UniProtKB database for higher plants contained such a highly charged fragment, although they were not identical (Supplementary Fig. S2A at *JXB* online). Additionally, the charged fragment also appeared in the ER-localized HSP90 from the unicellular green algae *Chlamydomonas reihhardtii*, but not the one from other mycota or fungal species such as *Dictyostelium discoideum*, *Blastocladiella emersonii* and *Coprinopsis cinerea* (Supplementary Fig. S2A). This suggested that the identified charged region may be specific to plants or those species that can perform photosynthesis.

**Fig. 2.**
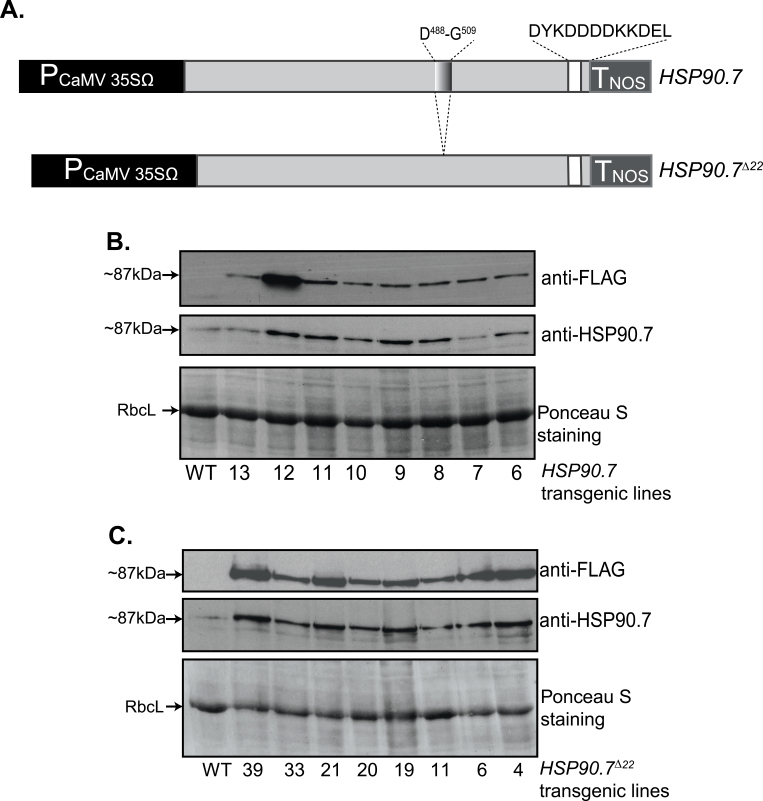
HSP90.7 constructs and screening of transgenic plants that express HSP90.7 or HSP90.7^Δ22^. (A) Schematic diagrams of HSP90.7 and HSP90.7^Δ22^ coding sequence constructs in binary vectors used for plant transformation. The charged fragment D488–G509 in HSP90.7 is deleted in HSP90.7^Δ22^. The C-terminal FLAG tag and ER-retention sequences are shown above. (B, C) Immunoblotting of transgenic plants expressing FLAG-tagged wild-type HSP90.7 (B) and HSP90.7^Δ22^ (C). Total proteins were prepared from 10-d-old T2 seedlings, resolved by 8% SDS-PAGE, and immunoblotted with anti-FLAG and anti-HSP90.7 antibodies.

We then modelled the HSP90.7 structure using 3D-JIGSAW (Bates *et al.*, 2001; Contreras-Moreira and Bates, 2002) and aligned the predicted structure with that of canine (*Canis familiaris*) GRP94 (CfGRP94) (Dollins *et al.*, 2007). As shown in Supplementary Fig. S2B, the charged region in the HSP90.7 middle domain seemed to form an extra loop compared with CfGRP94. To study the function of HSP90.7 *in vivo* and the possible role of this charged fragment, we made two HSP90.7 constructs: FLAG-tagged HSP90.7 and FLAG-tagged HSP90.7^Δ22^ in which the 22 aa charged region was deleted (Fig. 2A). For both constructs, the FLAG was added before the KDEL ER-retention motif to facilitate transgene detection without impairing ER localization.

### The highly charged region is required for *in vivo* HSP90 activity in resistance to ER stresses

**Fig. 3.**
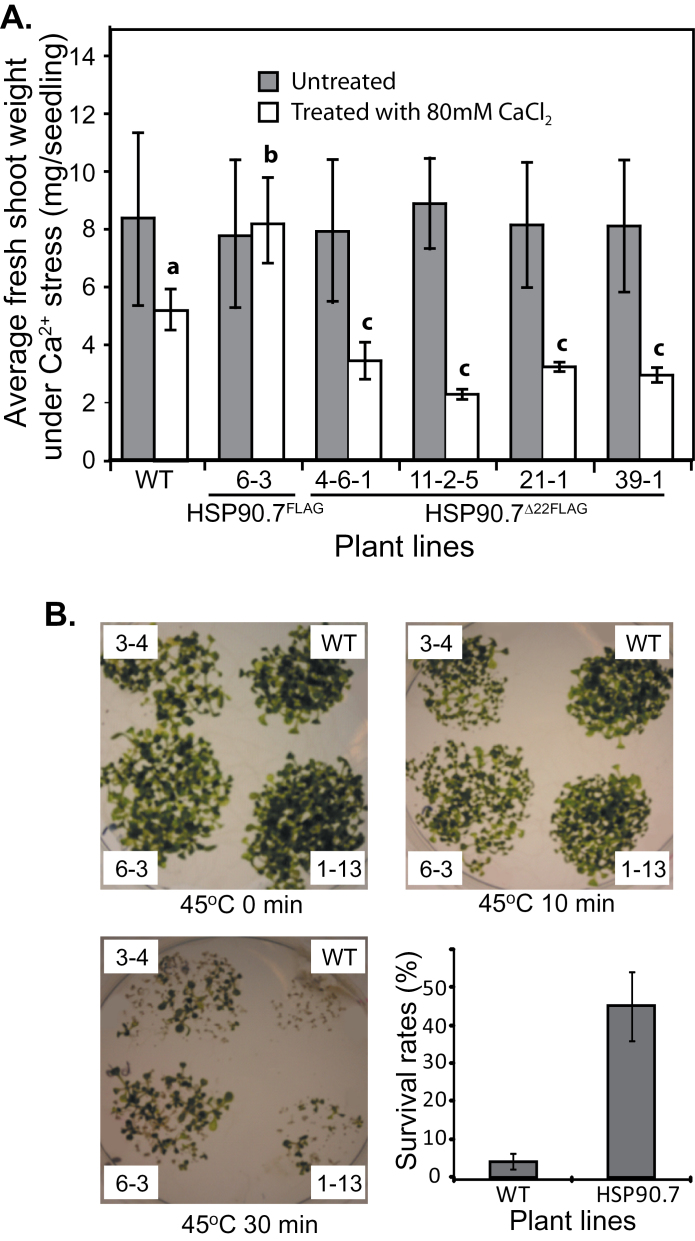
FLAG tag does not significantly affect HSP90.7 function *in vivo*. (A) High calcium resistance test for transgenic plants overexpressing FLAG-tagged HSP90.7 and HSP90.7^Δ22^. Seeds were germinated on ½ strength MS medium containing 80mM CaCl_2_ and grown for 3 weeks. The average fresh weight of seedlings was measured for wild-type Col-0 (WT), homozygous HSP90.7 overexpression line 6-3, and four homozygous HSP90.7^Δ22^ expression lines (4-6-1, 11-2-5, 21-1, and 39-1). Error bars represent standard deviation (SD) from at least 40 seedlings, and bars with different letters on top are significantly different as examined by *t*-test (*P*<0.05). (B) Heat-shock resistance test for transgenic plants overexpressing FLAG-tagged HSP90.7. Seeds were germinated on ½ strength MS medium for 6 d before heat-shock treatment for 5, 10, or 30min at 45 °C. Survival rates were calculated as the percentage of seedlings that had at least one green leaf after 6 d of recovery from 30min heat-shock treatment. The survival rates were averaged from three independent transgenic lines. Error bars represent SD.

To understand if the addition of a FLAG tag at the HSP90.7 C terminus affected *in vivo* chaperone functioning, transgenic overexpression lines were generated (Fig. 2B, C) and the homozygous progeny of overexpression lines (Supplementary Fig. S5 at *JXB* online) were first used to examine their tolerance to high calcium (Ca^2+^) and heat-shock stresses. Examination of homozygous line 6-3 indicated that HSP90.7 overexpression lines conferred better resistance to 80mM of CaCl_2_ compared with wild-type plants, which on average had 30% less fresh weight than overexpression lines (Fig. 3A). This agreed with a previous observation that overexpression of wild-type untagged HSP90.7 helps plants resist to high concentrations of Ca^2+^ stress (Song *et al.*, 2009*b*). Surprisingly, seedlings expressing HSP90.7^Δ22^ seemed to be significantly more sensitive to a high concentration of Ca^2+^, and resulted in much less fresh weight compared with wild-type seedlings as tested in all four independent homozygous transgenic lines (4-6-1, 11-2-5, 21-1, and 39-1) (Fig. 3A). To test if overexpression of FLAG-tagged HSP90.7 improved heat-shock resistance, transgenic seedlings grown for 6 d were heat shocked at 45 °C. It was noted that overexpression of FLAG-tagged HSP90.7 significantly improved the seedlings’ survival rate (Fig. 3B). Less than 5% of wild-type seedlings survived 30min of heat shock, whereas approximately 45% of seedlings that overexpressed *HSP90.7* survived 30min of heat shock. These results indicated that overexpression of HSP90.7 improves a plant’s heat-shock resistance, and that the FLAG tag does not seem to affect significantly the *in vivo* function of HSP90.7. However, similar assays were tested for seedlings expressing HSP90.7^Δ22^, and it was found that seedlings expressing HSP90.7^Δ22^ behaved similarly to wild-type seedlings and did not have better heat-shock resistance (Supplementary Fig. S6 at *JXB* online).

**Fig. 4.**
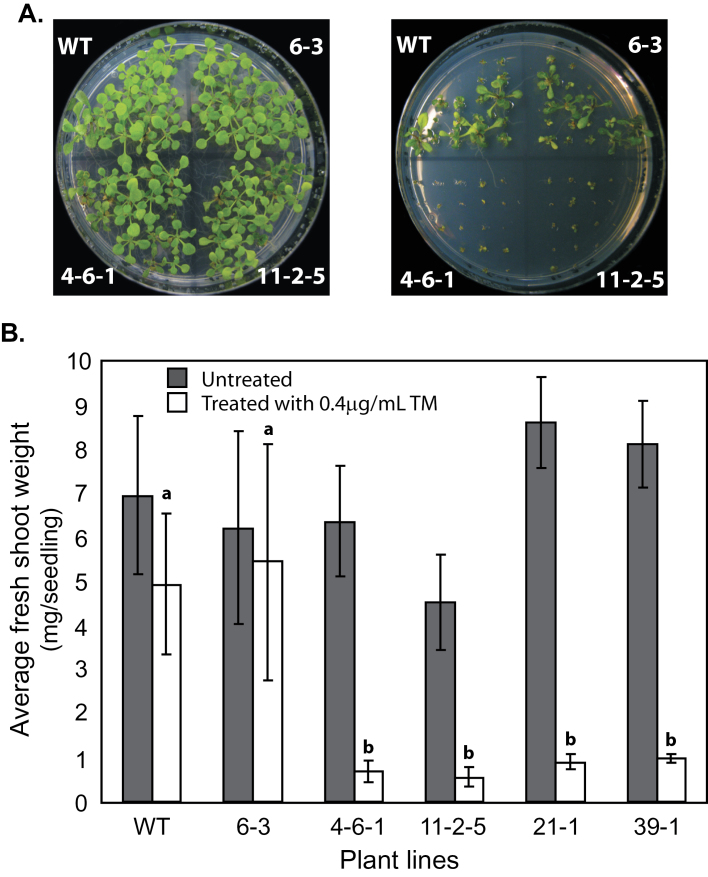
Expression of AtHSP90.7^Δ22^ shows a dominant-negative effect on ER stress resistance. (A) Left: seeds were germinated on ½ strength MS medium and grown for 11 d. Right: seeds were germinated on ½ strength MS medium containing 0.4 μg ml^–1^ tunicamycin for 3 d and then transferred to ½ strength MS medium for 11 d. WT represents wild-type Col-0 seedlings, 6-3 is a homozygous FLAG-tagged HSP90.7 expression line, and 4-6-1 and 11-2-5 are two examples of homozygous FLAG-tagged HSP90.7^Δ22^ expression lines. (B) Average fresh shoot weight per seedling. The fresh shoot weights were measured after 11 d of recovery as shown in (A). Four different homozygous FLAG-tagged HSP90.7^Δ22^ lines (4-6-1, 11-2-5, 21-1, and 39-1) and one FLAG-tagged HSP90.7 line (6-3) are shown. Three replicates were performed and error bars represent SD. Bars with different letters on top are significantly different as examined by *t*-test (*P*<0.05).

HSP90.7 is an ER-localized molecular chaperone, and ER stress usually results from improper protein homeostasis within the ER (Gardner *et al.*, 2013). To examine if overexpression of FLAG-tagged HSP90.7 conferred specific resistance to ER stress, we tested seedling growth on medium supplemented with tunicamycin, a commonly used reagent that specifically inhibits the first step of the *N*-glycosylation pathway within the ER (Duksin and Mahoney, 1982). It was noted, however, that seedlings overexpressing FLAG-tagged wild-type HSP90.7 had very similar average fresh weights to wild type after 11 d of recovery, indicating that overexpression of HSP90.7 did not significantly improve ER-specific stress resistance, at least under the tested conditions (Fig. 4A, B). Interestingly, seedlings expressing FLAG-tagged HSP90.7^Δ22^ had increased sensitivity to tunicamycin. None of the four tested homozygous expression lines recovered well under the tested conditions (Fig. 4A, B). Combined with the stress test with a higher Ca^2+^ concentration (Fig. 3A), this suggested that the 22 aa fragment in the middle domain is essential for proper HSP90.7 function *in vivo* and that deletion of this charged fragment caused a dominant-negative effect in both tunicamycin and high Ca^2+^ resistance. Nevertheless, a close look at the transgenic lines that expressed HSP90.7^Δ22^ did not reveal any significant growth defect at early or late development stages under normal growth conditions (16h photoperiod, 22 °C, 110 μmol m^–2^ s^–1^), by examining their leaf initiation, rosette radius, flowering, and flower organ development (Supplementary Fig. S3 at *JXB* online).

### Deletion of the highly charged sequence in the HSP90.7 middle domain does not affect its general molecular chaperone activity *in vitro*

**Fig. 5.**
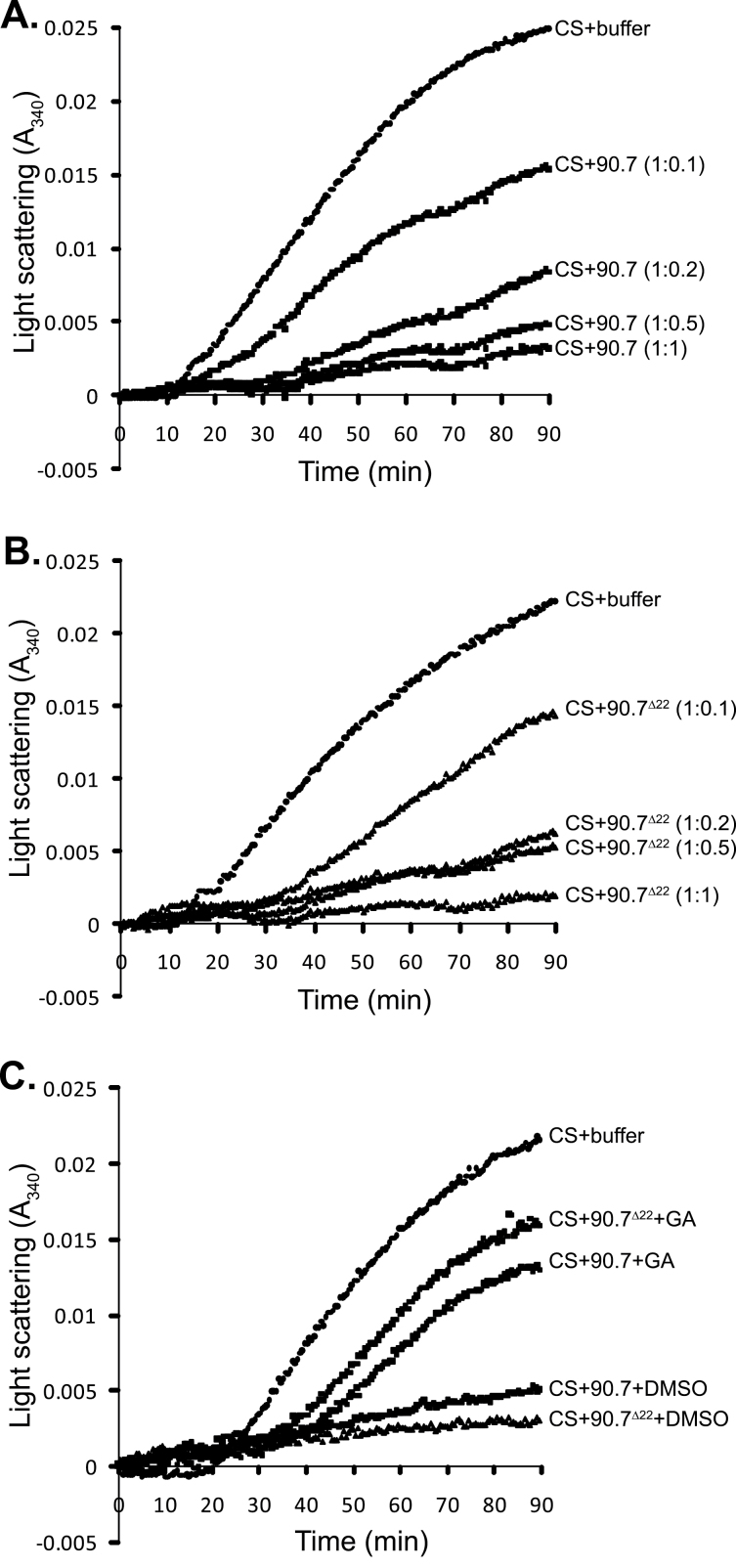
Chaperone activity of HSP90.7 and HSP90.7^Δ22^ *in vitro*. (A) Citric synthase (CS, 500nM) was incubated in the absence (+buffer) or presence of different amounts of HSP90.7 (+90.7). The molar ratios of CS:HSP90.7 are indicated in parentheses. (B) CS (500nM) was incubated in the absence (+buffer) or presence of different amounts of HSP90.7^Δ22^ (+90.7^Δ22^). The molar ratios of CS:HSP90.7^Δ22^ are indicated in parentheses. (C) CS (500nM) was incubated in the presence of equimolar HSP90.7 or HSP90.7^Δ22^ in the absence (+DMSO) or presence of 10 μM geldanamycin (+GA).

To understand why deletion of the charged region in the middle domain of HSP90.7 exerted a dominant-negative effect under ER-specific stress conditions, we examined the chaperone activity of HSP90.7 and HSP90.7^Δ22^ in preventing CS from heat-induced aggregation. The predicted mature forms of HSP90.7 and HSP90.7^Δ22^ were purified and mixed with CS. CS is an unstable protein that is denatured when heated at 45 °C and gradually aggregates; the amount of aggregate particles can be monitored by its light-scattering ability (CS+buffer in Fig. 5). When purified HSP90.7 or HSP90.7^Δ22^ was added to the CS mixture, both proteins were shown to inhibit heat-induced CS aggregation and the effect was concentration dependent (Fig. 5A, B). When equimolar amounts of HSP90.7 or HSP90.7^Δ22^ were added, heat-induced aggregation of CS was almost completely inhibited. There was no significant difference between HSP90.7 and HSP90.7^Δ22^ in preventing CS aggregation. As positive and negative controls, we also tested the effects of canine GRP94, and green fluorescent protein (GFP) on the inhibition of heat-induced aggregation of CS. Canine GRP94 was able to inhibit the aggregation of CS, while GFP was unable to inhibit the heat-induced aggregation of CS as expected (Supplementary Fig. S4 at *JXB* online). Interestingly, when geldanamycin, which tightly binds to the ATP-binding pocket of HSP90, was added, both HSP90.7 and HSP90.7 ^Δ22^ lost their ability to inhibit CS aggregation (Fig. 5C). These data indicated that deletion of the highly charged region in the HSP90.7 middle domain did not affect its *in vitro* chaperone activity and that the chaperone activity is probably conferred by a specific HSP90.7 conformation that can be blocked by the HSP90 inhibitor geldanamycin.

### Deletion of the highly charged fragment enhances the catalytic efficiency of HSP90.7 on ATP hydrolysis

**Fig. 6.**
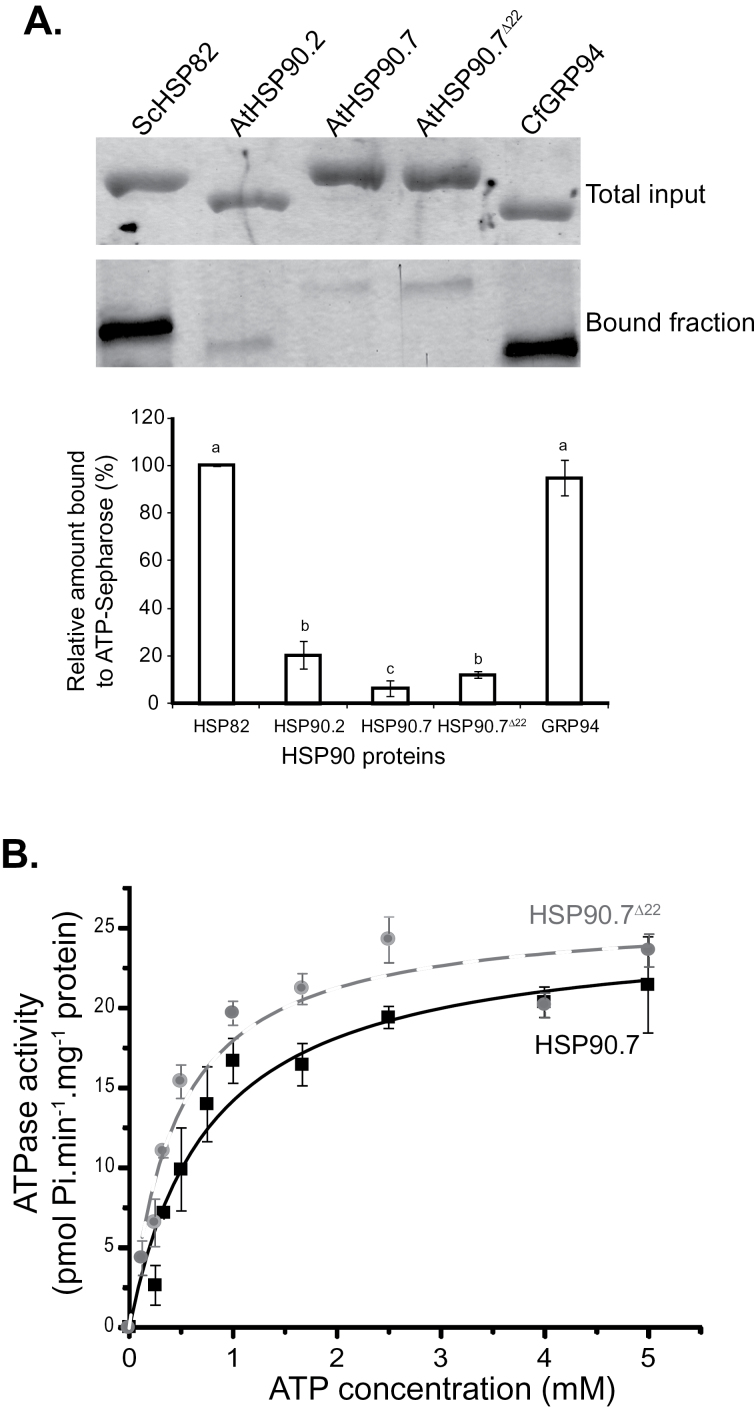
ATPase activities of HSP90.7 and HSP90.7^Δ22^. (A) ATP–Sepharose binding assay using yeast ScHSP82, *Arabidopsis* cytosolic AtHSP90.2, ER-localized AtHSP90.7 and AtHSP90.7^Δ22^, and canine CfGRP94. The total inputs and bound fractions were separated by 12% SDS-PAGE and visualized by silver staining (top two panels). The relative amounts of bound protein were scanned and analysed by Quantity One^®^ software (Bio-Rad), and are shown in the bottom panel. Bars with different letters on top indicate that they are significantly different as examined by *t*-test from three different repeats (*P*<0.05). (B) The kinetics of HSP90.7 and HSP90.7^Δ22^ in hydrolysing ATP. The specific activity was measured as geldanamycin-sensitive activity and error bars represent SD from at least three experimental repeats for each ATP concentration.

Since the functional cycle of HSP90 *in vivo* is a complex process that requires ATP binding and hydrolysis for both cytosolic HSP90s (Pearl and Prodromou, 2006; Taipale *et al.*, 2010) and mammalian GRP94 (Ostrovsky *et al.*, 2009), we then analysed the ATP-binding affinity of HSP90.7 together with the other representative HSP90 isoforms using an ATP–Sepharose binding assay. As shown in Fig. 6A, CfGRP94 and ScHSP82 had much higher ATP-binding affinity than *Arabidopsis* HSP90.2, HSP90.7 and HSP90.7^Δ22^ (top two panels of Fig. 6A). Close examination of the band intensity and quantitative analysis of the bands revealed that cytosolic *Arabidopsis* HSP90.2 had a slightly higher ATP-binding affinity than HSP90.7^Δ22^, while HSP90.7^Δ22^ had a higher ATP-binding affinity than the wild-type HSP90.7 (bottom panel of Fig. 6A).

The ATP-binding and hydrolysis activities for HSP90.7 and HSP90.7^Δ22^ were further analysed by NADH-coupled enzymatic assays and their kinetics were determined to be slightly different (Fig. 6B). Fitting the kinetics data to the Michaelis–Menton equation indicated that HSP90.7^Δ22^ had a similar turnover rate to wild-type HSP90.7 (Table 1), while the *K*_m_ value of HSP90.7^Δ22^ was only about 58% of that of the wild-type HSP90.7. This suggested that the deletion mutant HSP90.7^Δ22^ had a higher ATP-binding affinity, in agreement with the ATP–Sepharose binding assay (Fig. 6A), and as a result, the catalytic efficiency of HSP90.7^Δ22^ was almost twice as high as that of wild-type HSP90.7. Assays performed on ScHSP82 and CfGRP94 showed that they both had much lower *K*_m_ values compared with HSP90.7, albeit having much lower *k*_cat_ values (Table 1), thus also supporting the results obtained with the ATP–Sepharose binding assay (Fig. 6A)

**Table 1.** ATP hydrolysis activity of HSP90.7 and its homologues

HSP90 species	*k*_cat_ (min^–1^)	*K*_m_ (mM)	Catalytic efficiency (mM^–1^ min^–1^)
HSP90.7	2.26±0.17	0.77±0.18	2.94±0.91
HSP90.7^Δ22^	2.34±0.15	0.44±0.11	5.34±1.67
CfGRP94^*a*^	0.072±0.004	0.065±0.013	1.11±0.27
ScHSP82^*a*^	0.66±0.08	0.38±0.19	1.72±0.58

^*a*^ CfGRP94 represents GRP94 from *Canis familiaris* and ScHSP82 represents HSP82 from *Saccharomyces cerevisiae*.

### Expression of HSP90.7^Δ22^ does not induce an unfolded protein response (UPR) under normal growth conditions

**Fig. 7.**
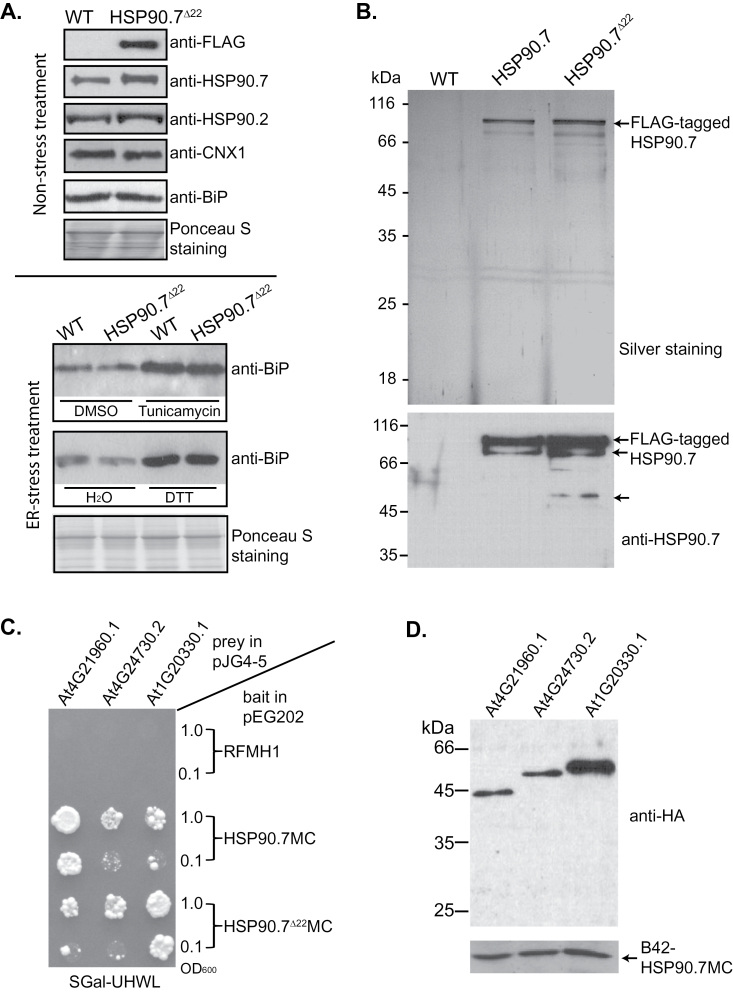
Immunoblotting of representative ER-localized chaperones and FLAG-tagged HSP90.7 complexes. (A) Top panel: total cell lysate of 2-week-old transgenic seedlings expressing HSP90.7^Δ22^ was immunoblotted with anti-FLAG, anti-HSP90.7, anti-HSP90.2, anti-calnexin, and anti-BiP antibodies. Bottom panel: 8-d-old seedlings grown on ½ strength MS agar plates were treated with 5 μM tunicamycin or 2mM DTT in liquid ½ strength MS salt for 8h, and the total proteins were then immunoblotted with anti-BiP antibody. Treatments with DMSO or H_2_O only were used as negative controls. (B) FLAG-tagged HSP90.7 or HSP90.7^Δ22^ complexes were purified using anti-FLAG antibody resin and resolved by 12% SDS-PAGE followed by silver staining (top). The bottom panel shows immunoblotting of the same samples with anti-HSP90.7 antibody. The arrows indicate the FLAG-tagged HSP90.7 or degradation intermediates. (C) Yeast EGY48 cells carrying prey and bait plasmids, which were originally diluted to an OD_600_ of 1.0 and 0.1 and applied to synthetic medium supplemented with galactose but without uracil, histidine, tryptophan, and leucine (SGal–UHWL), were grown for 7 d at 30 °C. pEG202-RFMH1 acted as a negative control. (D) Immunoblotting analysis of prey protein expression in yeast EGY48 cells carrying bait plasmid pEG202-HSP90.7MC and prey plasmids for At4G21960.1, At4G24730.2, and At1G20330.1, as indicated. Anti-HA antibody for prey protein and anti-HSP90.7 antibody for bait protein were used.

To further understand whether the enhanced sensitivity to tunicamycin and a high concentration of Ca^2+^ for HSP90.7^Δ22^ expression seedlings was due to excessive ER stress, we analysed the protein level of BiP, whose overexpression is a hallmark for ER stress (Gardner *et al.*, 2013). Interestingly, the expression level of BiP was not upregulated in FLAG-tagged HSP90.7^Δ22^ transgenic seedlings compared with wild type (Fig. 7A, top panel). The expression of cytosolic HSP90 or ER-chaperone calnexin was also not affected in transgenic HSP90.7^Δ22^ seedlings (Fig. 7A). This suggested that the general protein homeostasis in both the cytosol and the ER lumen might not be significantly affected under normal growth conditions. We also tested whether expression of HSP90.7^Δ22^ affected the UPR if treated with tunicamycin or DTT, the two commonly used UPR-inducing reagents. We noticed that both tunicamycin and DTT induced the expression of BiP well; however, there is no difference between wild type and HSP90.7^Δ22^ expression lines (Fig. 7A bottom panel), suggesting that expression of HSP90.7^Δ22^ might not significantly affect the normal UPR, at least not for the induction of BiP under ER stress. In an attempt to understand whether deletion of the charged region interfered with the association of any specific HSP90.7 clients, we purified both FLAG-tagged HSP90.7 and HSP90.7^Δ22^ complexes by affinity purification. Although both FLAG-tagged HSP90.7 and HSP90.7^Δ22^ were purified well, no other protein was significantly co-purified as shown by SDS-PAGE and silver staining (Fig. 7B). We tried to analyse the HSP90.7FLAG and HSP90.7^Δ22^FLAG protein complexes by liquid chromatography/tandem mass spectrometry; however, no robust HSP90.7 binding partners were identified (data not shown). This is presumably because client proteins bind HSP90.7 so weakly that the HSP90.7 protein complex was not well preserved in the affinity purification.

We further applied a yeast two-hybrid screening using the HSP90.7 middle and C-terminal domains as bait to identify potential HSP90.7 interactors with an *Arabidopsis* cDNA library (Tsai and Gazzarrini, 2012). The middle and C-terminal domains were chosen as bait because the middle domain is responsible for the client binding, and screening using these domains has proven successful to identify both co-chaperones and client proteins for cytosolic HSP90 isoforms (Zhao *et al.*, 2005). As a result, from over 100 colonies that grew on the selection medium, we identified seven potential HSP90.7 interactors, each of which were identified at least twice (Supplementary Table S1 at *JXB* online). Interestingly, analysis of three of the interactors, PRXR1 (At4G21960.1), the peroxidase 42, SMT2 (At1G20330.1), a sterol-C24-methyltransferase, and a calcineurin-like metallophosphoesterase (At4G24730.2), indicated that they all interacted with both wild-type and mutant HSP90.7MC but not with the negative-control protein (Fig. 7C). As a control, immunoblotting indicated that the three prey proteins, which all contained a signal peptide and are targeted to the ER during their *in vivo* synthesis, were expressed well in the test yeast cells (Fig. 7D). These data suggested that the charged region might not be required for client protein binding, at least not for the three tested candidates.

## Discussion

In this study, we analysed HSP90 family proteins that are located in different subcellular compartments of the cell. We particularly identified a short, charged region in the middle domain of plant ER-localized HSP90s, and this charged sequence was absent from any cytosolic, plastidic, mitochondrial, or metazoan-derived ER-localized HSP90 homologues (Fig. 1). Phylogenetic analyses have shown that GRP94 and cytosolic HSP90 family members evolved separately from a common ancestor, distinct from the ancestor of HtpG and TRAP (Chen *et al.*, 2006; Marzec *et al.*, 2012). The charged region in the middle domain is only observed in plant ER-localized GRP94 species, suggesting that this region may have evolved concurrently with the early symbiosis event from which cyanobacteria were harboured and had evolved into the chloroplast (Goksoyr, 1967; Osteryoung and Nunnari, 2003; Glynn *et al.*, 2007).

By analysing the ATP-hydrolysis activity, we found that deletion of the charged region in HSP90.7 middle domain resulted in a significantly higher ATP-hydrolysis efficiency due to increased ATP-binding affinity. The *k*_cat_ of HSP90.7 was comparable to that of HSP82 (0.566±0.080min^–1^) (Table 1), bacterial HptG (0.47min^–1^) (Panaretou *et al.*, 1998), and mitochondrial TRAP (0.1min^–1^) (Owen *et al.*, 2002), but much higher than that of human cytosolic HSP90 (0.04min^–1^) (McLaughlin *et al.*, 2002) and mammalian GRP94 (Table 1) (Dollins *et al.*, 2007). This suggests that plant ER-localized HSP90 species might better inherit the ATP-hydrolysis activity of its ancestor, the HptG group A protein, very early on in the formation of the eukaryotic cell (Chen *et al.*, 2006). Additionally, in terms of the mechanism of ATP hydrolysis, the higher catalytic activity of HSP90.7 suggests that it may generally adopt a catalytically active and closed conformation among apo, open and closed states (Southworth and Agard, 2008). It has been shown that the N terminus of HSP82 dimerizes and shifts to the closed conformation upon ATP binding, while the N terminus of GRP94 moves to a more open conformation, to which the nucleotide is more accessible (Dollins *et al.*, 2007). The open conformation does not allow efficient ATP hydrolysis, thus explaining the lower turnover rate of GRP94. Therefore, the ATP-hydrolysis mechanism of HSP90.7 may resemble the functional cycle of HSP82 and HptG, rather than GRP94 in mammals.

Reduced expression of HSP90.7 has been shown to repress CLV signalling activity (Ishiguro *et al.*, 2002). However, we did not observe any significant phenotype in HSP90.7 or HSP90.7^Δ22^ expression transgenic plants under normal growth conditions (Supplementary Fig. S3). One reason could be that either the enhancement or inhibition of the CLV signalling pathway by HSP90.7^Δ22^ expression did not reach a threshold that is necessary to trigger an observable phenotype. In agreement with this hypothesis, a previous study showed that *Arabidopsis* plants can tolerate up to a 10-fold high inducible CLV3 expression without displaying any phenotype (Muller *et al.*, 2006). Secondly, the previously observed phenotype associated with the *shd* mutant is from *Arabidopsis* ecotype Wassilewskija (Ishiguro *et al.*, 2002), and a similar *shd* mutant phenotype may not be observed in the Col-0 or Landsberg *erecta* (L*er*-0) background due to polymorphism of *CVL* genes.

Nevertheless, HSP90.7^Δ22^ transgenic plants in the Col-0 background showed a dominant-negative effect in resistance to tunicamycin and developed much less fresh weight during the recovery stage (Fig. 4), while overexpression of FLAG-tagged wild-type HSP90.7 did not induce such an effect. Because the expression of BiP or calnexin was not significantly induced (Fig. 7A), it is evident that the expression of HSP90.7^Δ22^ did not induce UPR in the ER. Additionally, we showed that HSP90.7^Δ22^ has a similar general chaperone activity in preventing CS from heat-induced aggregation (Fig. 5). Therefore, it is plausible to assume that the dominant-negative effect is not associated with the general chaperone activity of HSP90.7. We propose that there is a currently unknown mechanism that uniquely and sensitively monitors the altered ATPase activity of HSP90.7 and subsequently induces an HSP90.7-specific response in *Arabidopsis*. In agreement with this hypothesis, both ATP binding and hydrolysis for mammalian GRP94 have been shown to be essential for *in vivo* chaperone activity (Ostrovsky *et al.*, 2009). A recent study also showed that knockdown of GRP94 only selectively induces a subset of UPR-related chaperones, and such a response cannot be rescued by an ATPase-deficient GRP94 form (Eletto *et al.*, 2012), therefore leading the authors to propose that cells monitor the activity state of GRP94. We hypothesize that the enhanced ATP-binding and hydrolysis activity of HSP90.7^Δ22^, albeit by only 2-fold, may already signal that there is no need for the upregulation of certain specific ER-localized chaperones or foldases (Gupta and Tuteja, 2011) upon ER stress, thus leading to hypersensitivity, although the hallmark protein BiP is still upregulated (Fig. 7A).

We also observed that overexpression of HSP90.7 helped seedlings to resist a high concentration of Ca^2+^ and heat-shock stress. This agrees with previous studies on non-FLAG-tagged AtHSP90.7 (Song *et al.*, 2009*a*) and on an orchard grass ER-localized HSP90, which helps yeast resist heat-shock stresses (Cha *et al.*, 2009). Since the ER-localized HSP90 paralogue is one of the few abundant Ca^2+^-binding and storage proteins (Macer and Koch, 1988), overexpression of wild-type HSP90.7 may help Ca^2+^ storage and alleviate high Ca^2+^ toxicity. Surprisingly, expression of HSP90.7^Δ22^ did not help resistance against high Ca^2+^ stress and instead also exerted a dominant-negative effect, similar to that in tunicamycin resistance. The aforementioned mechanism that monitors the cellular HSP90.7 ATPase activity may also work in the Ca^2+^ stress and signalling pathway. It should be noted that, although the charged linker region between the N-terminal and middle domains binds Ca^2+^ and regulates cellular Ca^2+^ storage as shown for mammalian GRP94 (Biswas *et al.*, 2007), the role of the charged region in the HSP90.7 middle domain in directly modulating cellular Ca^2+^ binding and storage capacity requires further investigation. Finally, we screened a small set of HSP90.7 interactors (Supplementary Table S1) and demonstrated that the charged region in the HSP90.7 middle domain was not required for binding three of the candidate client proteins by a yeast two-hybrid assay (Fig. 7C). Our study therefore provides potential targets to further investigate the role of HSP90.7 in modulating cellular Ca^2+^ homeostasis and/or the tunicamycin resistance.

## Supplementary data

Supplementary data are available at *JXB* online.

Supplementary Fig. S1. N-terminal domain sequence alignment of ER-localized AtHSP90.7 with HSP90 homologues.

Supplementary Fig. S2. Amino acid sequence alignment and modelled 3D structure of HSP90.7 highlighting the highly charged region in the middle domain.

Supplementary Fig. S3. Transgenic plants expressing HSP90.7^Δ22^ do not show significant growth defects under normal growth conditions.

Supplementary Fig. S4. CfGRP94 prevents heat-induced aggregation of citrate synthase while purified GFP protein does not.

Supplementary Fig. S5. Immunoblotting of HSP90.7 proteins from homozygous transgenic lines.

Supplementary Fig. S6. Transgenic seedlings expressing HSP90.7^Δ22^ do not show better heat-shock resistance than wild type.

Supplementary Table S1. HSP90.7 interactors identified by yeast two-hybrid assay using the middle and C-terminal domains as bait.

Supplementary Data

## Abbreviations:

BiP: binding immunoglobulin protein
HSP90: heat-shock protein 90
ER: endoplasmic reticulum
FLAG: the 8 aa DYKDDDDK epitope
GFP: green fluorescent protein
GRP94: glucose-regulated protein 94
MS: Murashige and Skoog
CS: citrate synthase
SD: standard deviation
UPR: unfolded protein response.
