# Supplementary material for: A highly charged region in the middle domain of plant endoplasmic reticulum (ER)-localized heat-shock protein 90 is required for resistance to tunicamycin or high calcium-induced ER stresses
Source: J Exp Bot. 2014 Oct 8;66(1):113–24. doi: 10.1093/jxb/eru403 (PMC4265155; doi:10.1093/jxb/eru403)
Supplement: Supplementary Data [file supp_66_1_113__index.html]

A highly charged region in the middle domain of plant endoplasmic reticulum (ER)-localized heat-shock protein 90 is required for resistance to tunicamycin or high calcium-induced ER stresses — A highly charged region in the middle domain of plant endoplasmic reticulum (ER)-localized heat-shock protein 90 is required for resistance to tunicamycin or high calcium-induced ER stresses — Supplementary Data 

# A highly charged region in the middle domain of plant endoplasmic reticulum (ER)-localized heat-shock protein 90 is required for resistance to tunicamycin or high calcium-induced ER stresses

## Supplementary Data

Data files

**Files in this Data Supplement:**

- Supplementary Data - Supplementary Data
