# Supplementary material for: A highly charged region in the middle domain of plant endoplasmic reticulum (ER)-localized heat-shock protein 90 is required for resistance to tunicamycin or high calcium-induced ER stresses
Source: J Exp Bot. 2014 Oct 8;66(1):113–24. doi: 10.1093/jxb/eru403 (PMC4265155; doi:10.1093/jxb/eru403)
Supplement: Supplementary Data [file supp_eru403_jexbot129890_file001.pdf]

Supplementary materials

A highly charged region in the middle domain of plant ER-localized HSP90 is required for resistance to tunicamycin or high calcium-induced ER stresses

Lisa P. Chong, Yao Wang, Nanette Gad, Nathaniel Anderson, Bhavank Shah and Rongmin Zhao

Supplementary Table S1: HSP90.7 interactors identified by yeast two-hybrid using the middle and C-terminal domains as bait

Number	Locus	Gene model	Number of appearance	Protein name	Protein full length (AA)	Protein expressed in prey plasmid	Subcellular localization	Description
1	AT2G30140	AT2G30140.2	3	UGT87A2	455	C-terminal 255 amino acids	cytoplasm, cytosol, nucleus	Encodes a putative glycosyltransferase. Regulates flowering time via FLOWERING LOCUS C.
2	AT4G08330	AT4G08330.1	2	-	164	C-terminal 153 amino acids	nucleus, plasma membrane	unknown protein
3	AT4G21960	AT4G21960.1	3	PRXR1	330	C-terminal 214 amino acids	Extracellular region, membrane	Peroxidase 42
4	AT4G24780	AT4G24780.2	2		408	C-terminal 286 amino acids	Extracellular region, membrane	Pectin lyase-like superfamily protein
5	AT4G16760	AT4G16760.2	6	ACX1	664	C-terminal 211 amino acids	cytoplasm, peroxisome, plasmodesma	Defense response to fungus, insect; fatty acid metabolic process
6	AT4G24730	AT4G24730.2	2		311	N-terminal 280 amino acids	endomembrane	Calcineurin-like metallo-phosphoesterase superfamily protein;
7	AT1G20330	AT1G20330.1	2	SMT2	361	C-terminal 272 amino acids	Golgi apparatus, endoplasmic reticulum	Encodes a sterol-C24-methyltransferases involved in sterol biosynthesis.

Supplementary Figure S1

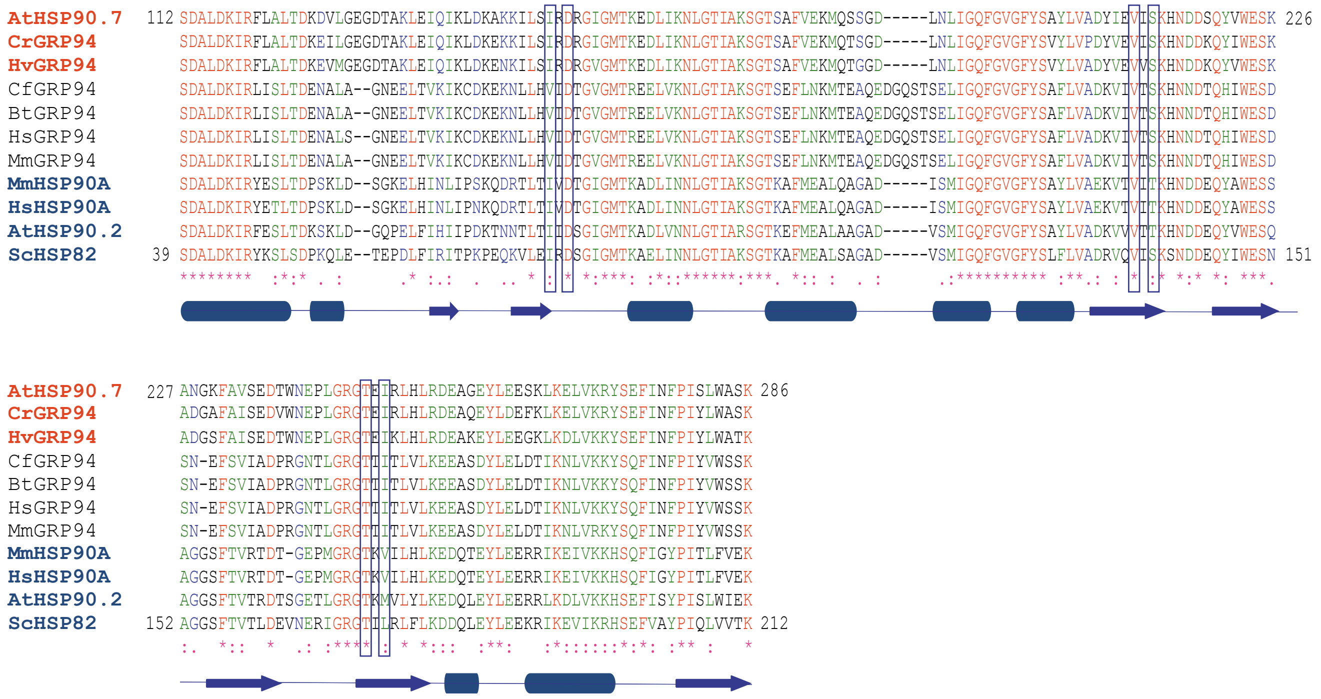

Figure S1. Sequence alignment of ER-localized AtHSP90.7 with HSP90 homologues localized in cytosol (blue), mammalian ER (black) and the ER of other plant species (red). Only partial N-terminal sequences (The positions of amino acids for AtHSP90.7 and yeast *Saccharomyces cerevisiae* HSP82, ScHSP82, are shown as references) are shown. Some conserved amino acids responsible for ATP binding (I77, D79, V136, S138, T171 and L173) are highlighted in blue rectangle boxes. The secondary structures (helices as cylinders, β-strands as arrows) are shown under the N-termimal sequences based on the structure of ScHSP82. AtHSP90.2: Arabidopsis cytosolic HSP90.2, HsHSP90A: human Hsp90 alpha, MmHSP90A: mouse HSP90A, MmGRP94: mouse GRP94, HsGRP94: human GRP94, BtGRP94: bovine GRP94, CfGRP94: canine GRP94, HvGRP94: Barley ER-localized HSP90, CrGRP94: Periwinkle ER-localized Hsp90.

Supplementary Figure S2.

A.

	473 ... 519	
A. thaliana	RKALDMIRKL AEEDPDEIHD DEKKDVEKSG EN.DEKKGQY TKFWNEFG	Plants
H. vulgare	RKALDMIRKL AEEDPDEYSN KEKTDDE.KS AM.EEKKGQY AKFWNEFG	
D. glomerata	RKALDMIRKL AEEDPDEYSN KEKTDEE.KS AM.EKKRGQY AKFWNEFG	
O. sativa	RKALDMIRKL AEEDPDEYSN KDKTDEE.KS AM.EEKKGQY AKFWNEFG	
Z. mays	RKALDMIRKL AEEDPDEYSN KDKTDEE.KS EM.EEKKGQY AKFWNEFG	
C. roseus	RKALDMIRKI ADEDPDEAND KDKKEVEEST DN.DEKKGQY AKFWNEFG	
V. vinifera	RKALDMIRKI ADEDPDESND KDKKDVEKSS DD.DEKKGQY AKFWNEFG	
P. trichocarpa	RKALDMIRKI ADEDPDEAND KDKKDVENSS D..DEKKGQY AKFWNEFG	
R. communis	RKALDMIRKI ADEDPDEMKE .DKKDVEDSG D..DEKKGQY AKFWNEFG	
X. viscosa	RKALDMIRKI ADEDPD...E SDKDHSEEAG EE.NEKKGLY TKFWNEFG	
P. taeda	RKALDMIRRI AEEDLDESDA KGKTDAESES EPDTEKKGKY VKFWNEFG	
C. reinhardtii	RKVLDMIRKM AEAEVKCKEM EEKGETEDKP SE..KECGQY AKFWEQFG	Green Algae
D. discoideum	VKFISMIKEL SED....... ....EDKTKY NEFFKKFG	Mycota
B. emersonii	SKTLQMFKTL AR........ ....NATSY EPLYKAYA	Fungi
C. cinerea	KRLIQLFAKL EK........ ....EEPRKW EKFQKTYG	
H. sapiens	RKTLDMIKKI AD........ ....DKYN. DTFWKEFG	Mammals
M. fascicularis	RKTLDMIKKI AD........ ....DKYN. DTFWKEFG	
P. abelii	RKTLDMIKKI AD........ ....DKYN. DTFWKEFG	
R. norvegicus	RKTLDMIKKI AD........ ....EKYN. DTFWKEFG	
M. musculus	RKTLDMIKKI AD........ ....EKYN. DTFWKEFG	
M. auratus	RKTLDMIKKI AD........ ....EKYN. DTFWKEFG	
O. cuniculus	RKTLDMIKKI AD........ ....EKYND DTFWK..G	
S. scrofa	RKTLDMIKKI AD........ ....EKYN. DTFWKEFG	
C. familiaris	RKTLDMIKKI AD........ ....EKYN. DTFWKEFG	
B. taurus	RKTLDMIKKI AD........ ....EKYN. DTFWKEFG	
G. gallus	RKTLDMIKKI AE........ ....EKYN. DTFWKEFG	

ER-localized Isoforms

A. thaliana	RKTFDMIQEI SES....... ....ENKEDY KKFWENFG	Chloroplastic Isoforms
S. moellendorffii	RKSFDLLDEI A......... ....NREKKEDY KIFWTCFS	
C. reinhardtii	RRSIEMLEEL AGK....... ....EGGEDY KTFWEAFG	
O. lucimarinus	RKTFDMLRDI AARE...... ....GDDY DTFWENFG	
A. thaliana	RKAFDMILGI SL........ ....SENREDY EKFWDNFG	Mitochondrial Isoforms
O. lucimarinus	KRVLKFLDEK AK........ ....KEPEKY NKWFANLG	
H. sapiens	QRLIKFFID. .... .QSKKDAEKY AKFFEDYG	
D. melanogaster	TRVIRFLQER S......... ....KKQPEEY EAFYRDYG	
A. thaliana	....KEDY NKFYEAFS	Cytosolic Isoforms
H. vulgare	KKCIELFFEI A......... ....ENKEDY NKFYEAFS	
H. sapiens	KKCLELFSEL AE........ ....DKENY KKFYEAFS	
D. melanogaster	KKTMELIEEL TE........ ....DKENY KKFYDQFS	

B.

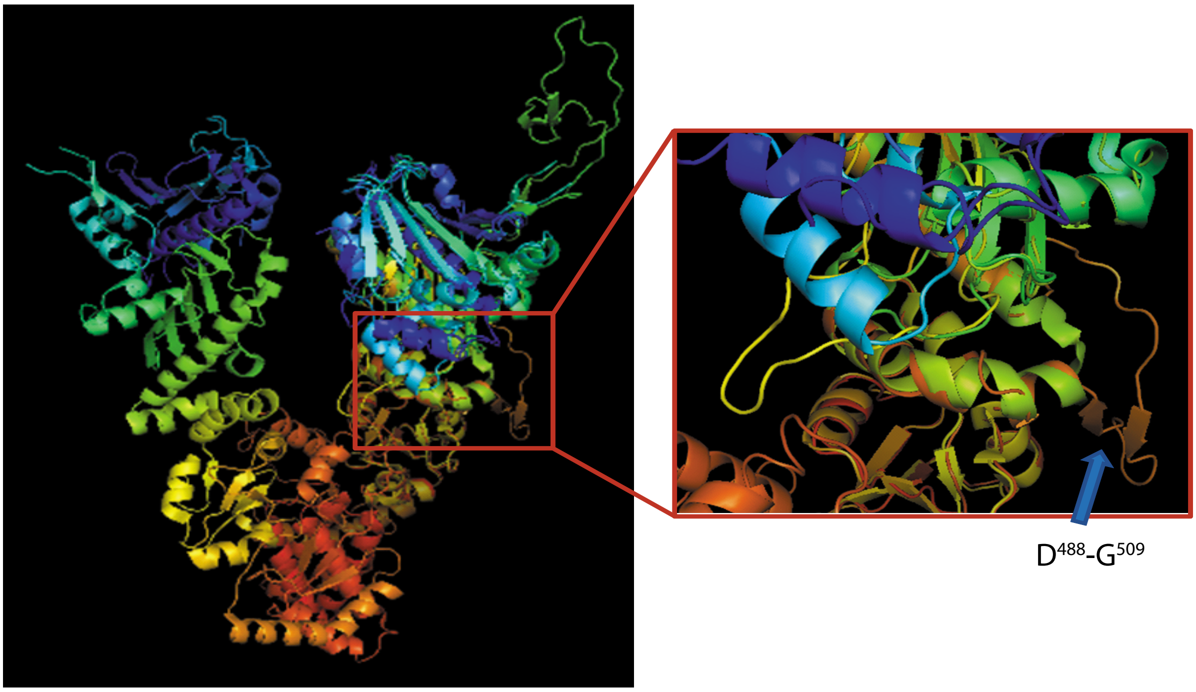

Figure S2. Sequence alignment of HSP90s surrounding the highly charged region of HSP90.7

A. HSP90 sequences include those from plants**:** *A. thaliana: Arabidopsis thaliana* (Mouse-ear cress) Q9STX5; *H. vulgare: Hordeum vulgare* (Barley) P36183; *D. glomerata: Dactylis glomerata* (Orchard grass) B7SA66; *O. sativa: Oryza sativa* (Rice) Q9MB32; *Z. mays: Zea mays* (Maize) B6U0V6; *C. roseus: Catharanthus roseus* (Madagascar periwinkle) P35016; *V. vinifera: Vitis vinifera* (Grape) E0CQ80; *P. trichocarpa: Populus trichocarpa* (Western balsam poplar) B9H4Z6; *R. communis: Ricinus communis* (Castor bean) B9R8A7; *X. viscosa: Xerophyta viscosa* Q8H6B6; *Pinus taeda* (Loblolly pine) A7YAU9; Green Algae **-** *C. reinhardtii: Chlamydomonas reinhardtii* A8I7T1; Mycota **-** *D. discoideum: Dictyostelium discoideum* (Slime mold) Q9NKX1; Fungi **-** *B. emersonii: Blastocladiella emersonii* (Aquatic fungus) B1NK18; *C. cinerea: Coprinopsis cinerea* (Inky cap fungus) D6RMR7; Mammals **-** *H. sapiens: Homo sapiens* (Human) P14625; *M. fascicularis*: *Macaca fascicularis* (Crab-eating macaque) Q4R520; *P. abelii: Pongo abelii* (Sumatran orang-utan) Q5R6F7; *R. norvegicus: Rattus norvegicus* (Rat) Q66HD0; *M. musculus*: *Mus musculus* (Mouse) P08113; *M. auratus*: *Mesocricetus auratus* (Golden hamster) P08712; *O. cuniculus: Oryctolagus cuniculus* (Rabbit) O18750; *S. scrofa*: *Sus scrofa* (Pig) Q29092; *C. familiaris*: *Canis familiaris* (Dog) P41148; *B. taurus*: *Bos taurus* (Bovine) Q95M18; *G. gallus*: *Gallus gallus* (Chicken) P08110; Chloroplastidic HSP90s **-** *A. thaliana: Arabidopsis thaliana* (Mouse-ear cress) Q9SIF2; *S. moellendorffii: Selaginella moellendorffii* (Gemmiferous spikemoss) D8R9C5; *C. reinhardtii: Chlamydomonas reinhardtii* (Green algae) Q66T67; *O. lucimarinus: Ostreococcus lucimarinus* (Green algae) A4RQD9; Mitochondrial HSP90s **-** *A. thaliana: Arabidopsis thaliana* (Mouse-ear cress) F4JFN3; *O. lucimarinus: Ostreococcus lucimarinus* (Green algae) A4S3C8; *H. sapiens: Homo sapiens* (Human) Q12931; *D. melanogaster: Drosophila melanogaster* (Fruit fly) A1Z6L9; and from Cytosolic HSP90s **-** *A. thaliana*: *Arabidopsis thaliana* (Mouse-ear cress) Q8GRU8; *H. vulgare*: *Hordeum vulgare* (Barley) Q7XJ80; *H. sapiens: Homo sapiens* (Human) P08238; *D. melanogaster: Drosophila melanogaster* (Fruit fly) P02828

B. The structure of HSP90.7 which was modelled using 3Djigsaw based on the GRP94 structure (2o1v). The predicted HSP90.7 structure is superimposed into the structure of GRP94 structure using PyMol. The 22-amino-acid plant-specific sequence of HSP90.7 (D488-G509) forms a loop in the middle domain (arrow).

Supplementary Figure S3

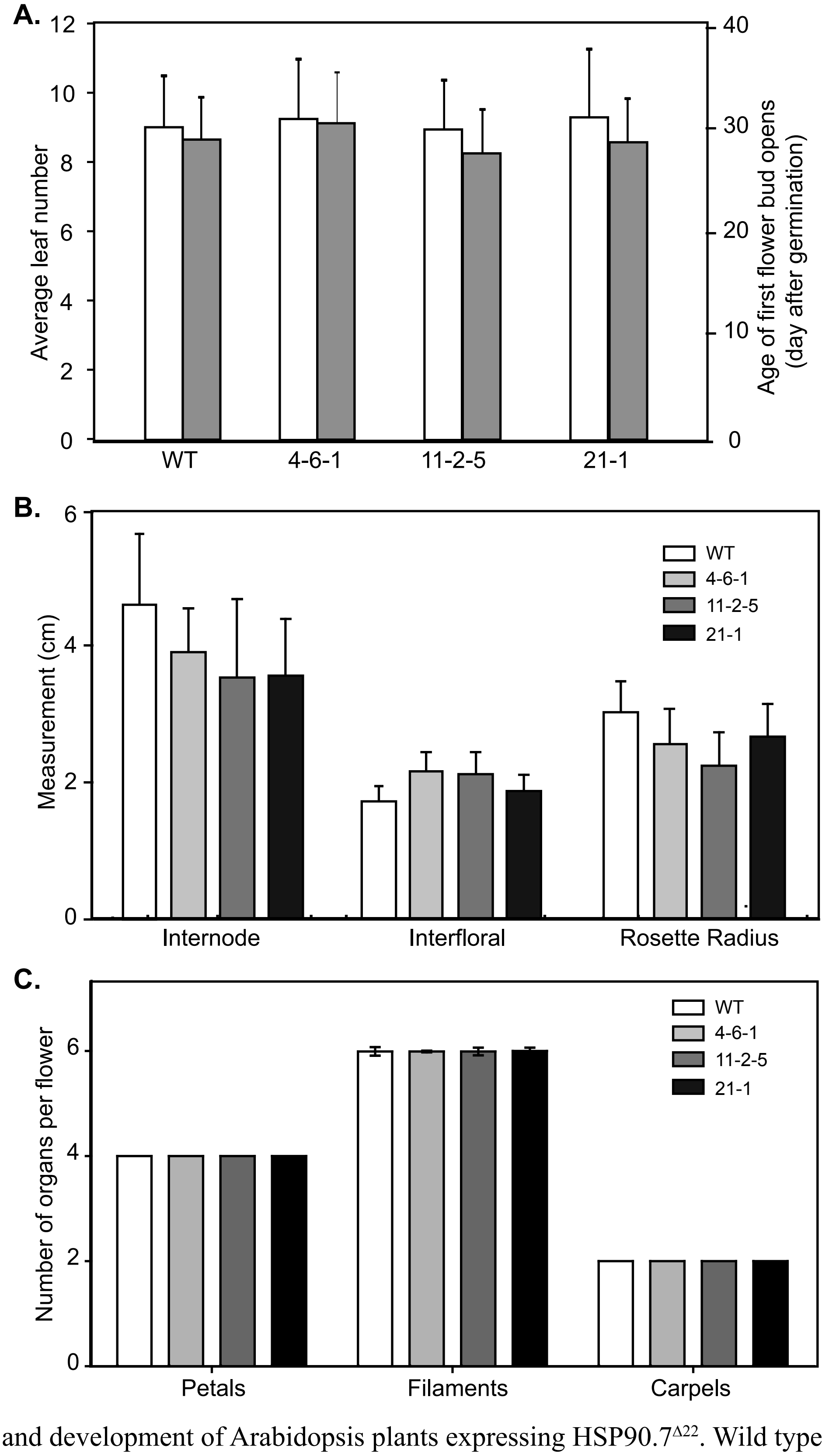

Figure S3. Growth and development of Arabidopsis plants expressing HSP90.7$^{\Delta22}$. Wild type (WT) and three independent transgenic HSP90.7$^{\Delta22}$ homozygote plants (4-6-1, 11-2-5 and 21-1) were germinated on MS medium under illumination of 120 µmol.s^{-1}.m^{-2} at 22°C with 16h/8h light/dark photoperiod. The seedlings were transferred to soil at 10 days old and continuously grown under 110 µmol.s^{-1}.m^{-2} at 22°C with 16h/8h light/dark photoperiod.

A. Total vegetative rosette leaves before bolting (white bar) and age (day after germination) when the first flower bud opens (grey bar). The data represent the average and standard deviation from at least 15 plants for each line.

B. The average lengths of internode (for all cauline leaves from the primary influorescence), interfloral (for the first 5 flowers on primary influorescence) and rosette radius measured at the day of first flower opens. At least 10 different plants were examined for each line.

C. The average number of petals, filaments and carpels examined for the first 10 flowers on primary influorescence. At least 15 plants were examined for each line. It should be noted that no error bar is shown for petal and carpel data becasue of no obvious variation of the petal or carpel numbers.

Supplementary Figure S4

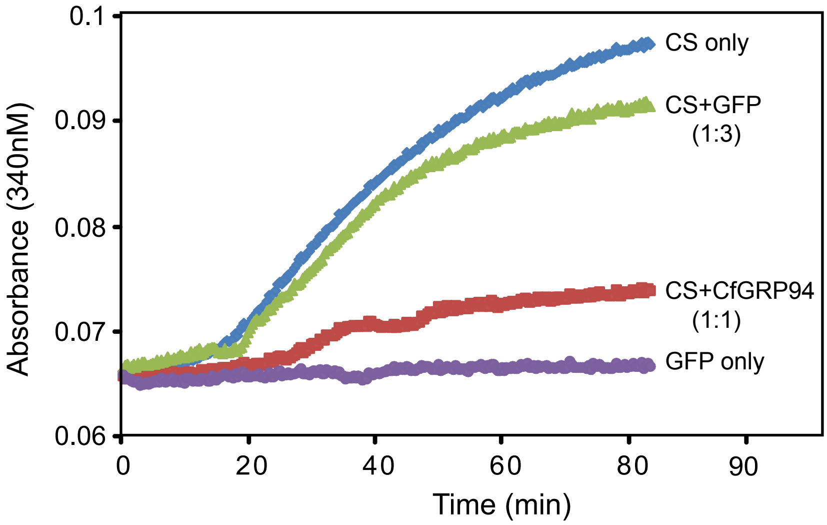

Figure S4. Chaperone activity of canine GRP94 on citrate cynthase (CS). CS (500 nM) was incubated in the absence (CS only) or presence of green fluorescent protein (GFP) in a molar ration of 1:3 (CS :GFP), or equal molar of canine GRP94 (CfGRP94). GFP protein only was also analyzed as a control. His6-tagged GFP was expressed and purified from *E. coli*.

Supplementary Figure S5

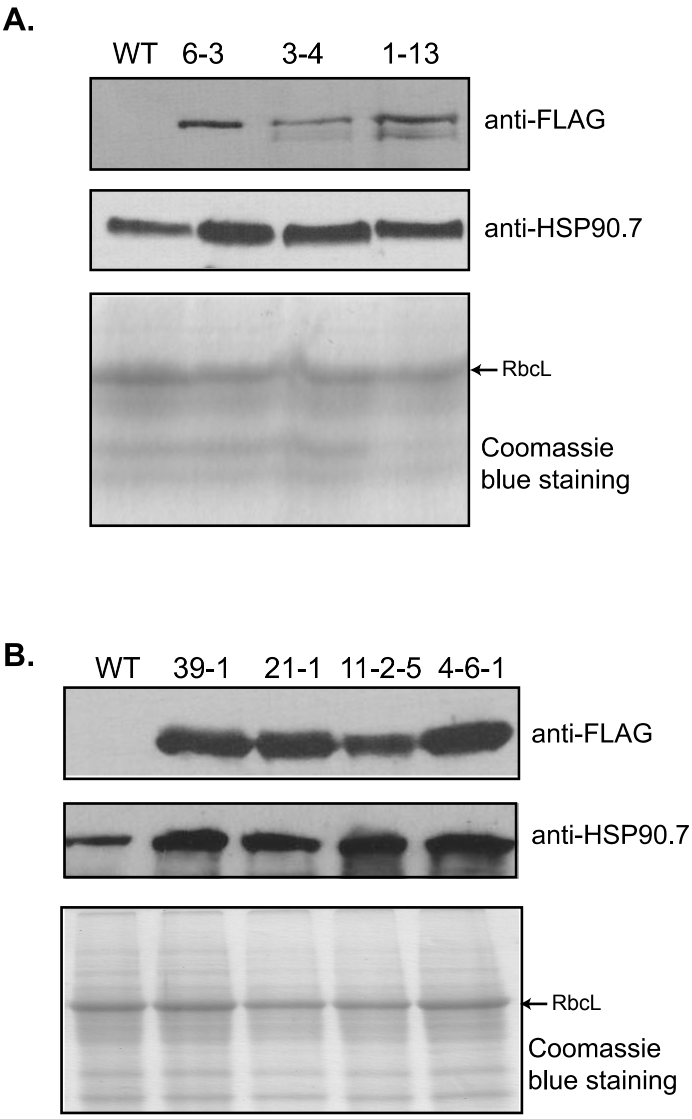

Figure S5. Immunoblotting of total cell lysate proteins from homozygote lines with anti-FLAG and anti-HSP90.7 antibodies. 10 μg of total soluble proteins from 8-day-old seedlings were separated on 8% SDS-PAGE, transferred to nitrocellulose membrane and immunoblotted with anti-FLAG (top panel), anti-HSP90.7 (middle panel). 12% SDS-PAGE gels stained with Coomassie blue are shown in bottom panels. WT represents untransformed seedlings.

A. Homozygote lines expressing FLAG-tagged HSP90.7.

B. Homozygote lines expressing FLAG-tagged HSP90.7$^{\Delta22}$.

Supplementary Figure S6

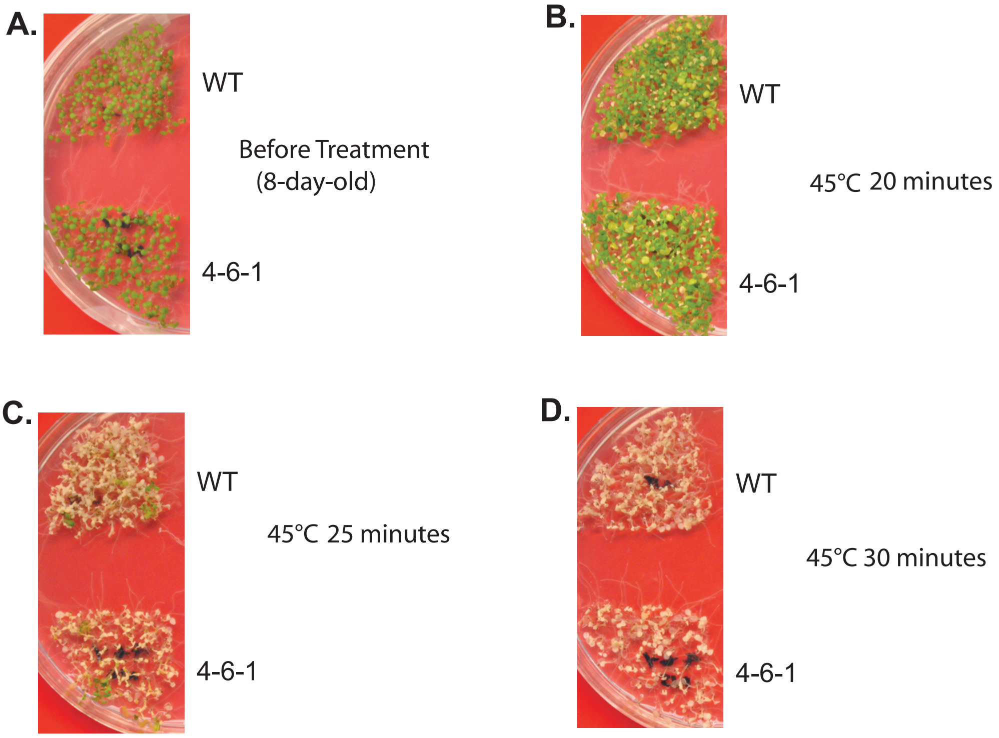

Figure S6. 8-day-old seedlings grown on 1/2 MS medium were heat shocked at 45˚C for 20 min (B), 25 min (C), or 30 min (D) and then recovered at 22˚C, 16h light photoperiod with illumination of 110 µmol.m^{-2}.s^{-1} for 7 days. 8-day-old seedlings before heat shock (A) are shown to indicate equal growth at the beginning. WT and 4-6-1 represent wild type and homozygote seedlings expressing HSP90.7$^{\Delta 22}$. Only one set of representative data are shown.
